# Barriers and Facilitators to Implementing Bundled Acupuncture and Yoga Therapy to Treat Chronic Pain in Community Healthcare Settings: A Feasibility Pilot

**DOI:** 10.1089/acm.2020.0394

**Published:** 2021-06-16

**Authors:** Belinda J. Anderson, Paul Meissner, Donna M. Mah, Arya Nielsen, Steffany Moonaz, M. Diane McKee, Benjamin Kligler, Mirta Milanes, Hernidia Guerra, Raymond Teets

**Affiliations:** ^1^ College of Health Professions, Pace University, New York, NY, USA.; ^2^ School of Nursing and Health Studies, Monmouth University, West Long Branch, NJ, USA.; ^3^ Department of Family and Social Medicine, Albert Einstein College of Medicine, Bronx, NY, USA.; ^4^ Pacific College of Health and Science, San Diego, CA, USA.; ^5^ Department of Family Medicine and Community Health, Icahn School of Medicine at Mount Sinai, New York, NY, USA.; ^6^ Maryland University of Integrative Health, Laurel, MD, USA.; ^7^ Family Medicine and Community Health, University of Massachusetts Medical School, Worcester, MA, USA.; ^8^ Integrative Health Coordinating Center, US Veterans Health Administration, Washington, DC, USA.; ^9^ Institute for Family Health, New York, NY, USA.

**Keywords:** implementation, acupuncture therapy, yoga therapy, nonpharmacologic pain care, underserved setting

## Abstract

***Objective:*** To identify factors associated with implementing bundled group acupuncture and yoga therapy (YT) to treat underserved patients with chronic pain in community health center (CHC) settings. This is not an implementation science study, but rather an organized approach for identification of barriers and facilitators to implementing these therapies as a precursor to a future implementation science study.

***Design:*** This study was part of a single-arm feasibility trial, which aimed to test the feasibility of bundling GA and YT for chronic pain in CHCs. Treatment outcomes were measured before and after the 10-week intervention period. Implementation feasibility was assessed through weekly research team meetings, weekly yoga provider meetings, monthly acupuncture provider meetings, and weekly provider surveys.

***Settings:*** The study was conducted in New York City at two Montefiore Medical Group (MMG) sites in the Bronx, and one Institute for Family Health (IFH) site in Harlem.

***Subjects:*** Participants in the feasibility trial were recruited from IFH and MMG sites, and needed to have had lower back, neck, or osteoarthritis pain for >3 months. Implementation stakeholders included the research team, providers of acupuncture and YT, referring providers, and CHC staff.

***Results:*** Implementation of these therapies was assessed using the Consolidated Framework for Implementation Research. We identified issues associated with scheduling, treatment fidelity, communication, the three-way disciplinary interaction of acupuncture, yoga, and biomedicine, space adaptation, site-specific logistical and operational requirements, and patient-provider language barriers. Issues varied as to their frequency and resolution difficulty.

***Conclusions:*** This feasibility trial identified implementation issues and resolution strategies that could be further explored in future implementation studies.

Clinical Trial Registration No.: NCT04296344.

## Introduction

Evidence-based guidelines are slow to be implemented in medicine,^1^ including evidence-based nonpharmacologic strategies for pain, a high priority in the United States due to the opioid crisis.^2–4^ Despite consistent efforts and significant research funding to mitigate opioid prescribing, almost 70%^5^ of the 67,367 drug overdose deaths in the United States^6^ in 2018 were due to opioids. The prevalence of chronic pain problems in the general adult U.S. population is high^7^; estimates have ranged from 10% to 40% in recent large surveys.^8–11^

Acupuncture therapy is effective for chronic pain conditions,^12–14^ including chronic low back pain,^15–18^ neck pain,^18–20^ shoulder pain, and knee pain from osteoarthritis.^21–27^ Yoga therapy (YT) is beneficial for back^28^ and neck pain,^29^ osteoarthritis,^30^ rheumatoid arthritis,^31^ and fibromyalgia,^32^ in addition to pain-associated function^28^ and disability.^33^ Both acupuncture therapy and YT have positive safety profiles and low risk of adverse events.^7^

Underserved populations are disproportionately affected by chronic pain^9,34,35^ and the opioid crisis.^36^ Access to nonpharmacologic pain care is limited as is insurance coverage,^19^ To evaluate an access model, we recently conducted a trial comparing group acupuncture (GA) verses individualized acupuncture for chronic pain in an underserved patient population.^37^ GA was effective for chronic pain and highly acceptable to patients^38,39^ GA, which involves treating two to four seated patients at the same time in an open setting where patients can interact, is less expensive than individualized acupuncture involving one practitioner and one patient in a private room.

Acupuncture is a passive therapy that also encourages patient engagement and activation. As patients with chronic pain improve there is a natural progression to want and need to increase activity and movement recovery. YT is an active therapy with proven benefits in musculoskeletal pain disorders and pain-associated disability. Both therapies have been studied in underserved settings.^37,40^ With an aim toward assessing the benefit and implementation issues associated with bundling GA and YT for chronic pain we undertook a feasibility study.^41^ YT was used instead of yoga classes because YT is delivered one-on-one or in small groups, includes a thorough client intake, an individualized plan of care, and ongoing assessment of progress.^42^ This is much better suited for people with chronic pain and related multimorbidities. The study was undertaken in three community health centers (CHCs) in New York City.

For research consistency and replicability, we used a modified Delphi process to develop treatment manuals.^43,44^ Consensus manualization provides guidelines for patient treatment, allowing for trial practitioners to be ‘responsive’ to real world heterogeneous and evolving patient presentations. The effectiveness of the combined GA and YT was measured using a range of outcomes instruments. Primary outcomes were pain interference and pain intensity. Secondary outcomes were pain-free days, depression, functional status, patient activation, and pain medication utilization.^37^ These were used during preintervention usual care, and up to 24 weeks after the end of treatment. Results are currently in analysis.

Although this was not an implementation science study the Consolidated Framework for Implementation Research (CFIR)^45^ was utilized to best understand the implementation factors associated with this feasibility study. Exploration of these factors was assessed using the five domains of the CFIR structure—characteristics of the intervention, inner setting, outer setting, individuals involved, and implementation process. We also describe the tools and methods used to collect implementation process data, and how this data informed our understanding of strategies to address limitations and barriers to implementation. Although implementation science is increasingly recognized as important to the translation of research to clinical practice, very little research has been undertaken on the implementation of complementary and integrative therapies (CIH). Our study therefore represents an important addition to the literature and a precursor to future implementation science studies.

## Methods

Our feasibility trial was approved by the Institutional Review Boards at Albert Einstein College of Medicine, Icahn School of Medicine at Mount Sinai, and the Institute for Family Health (IFH). The trial was registered with ClinicalTrials.gov number NCT04296344.

### The feasibility trial

#### Settings

The multisite collaborative trial was conducted at three CHCs—two in the Montefiore Medical Group (MMG)—the Family Health Center, and Williamsbridge Family Practice Center in the Bronx, and the other in the IFH—Family Health Center of Harlem. All three are federally qualified health centers, funded through the Health Resources and Services Administration to provide primary care in underserved areas.^46^

#### Study participants

Study participants were recruited from the IFH and MMG sites. Participants needed to have had neck, lower back, or osteoarthritis pain for >3 months.

#### Interventions

The trial was designed such that participants received GA therapy first. GA sessions were staffed with one acupuncturist who could initiate treatment of up to four participants per hour.^41^ The acupuncture therapy intervention, outlined in the manual,^44^ consisted of acupuncture needling and a range of other East Asian medicine modalities including Gua Sha, Tui Na, and auricular therapy with needles and/or ear seeds. Acupuncture sessions lasted from 15 to 40 min, with the timing often adapted to facilitate participants starting their YT session.

During the first 2 weeks of the intervention, participants only received GA. On week 2, participants received a yoga evaluation, with participants starting YT immediately following acupuncture on week 3. YT sessions were 30–35 min and were either conducted individually or in dyads (two participants undertaking the therapy together with one yoga therapist). Use of dyads was decided to facilitate the timing of participants completing GA, with less wait time between the acupuncture and yoga therapies. The yoga manual provided categories of practices for the yoga providers to choose from, which included breathing practices, poses for strengthening, mobilizing, and balancing, relaxation practices, and lifestyle practices. The choice of yoga positions (asanas) was based on those used in prior chronic pain yoga research.^47–50^ A detailed description of dyad YT that was developed for this trial, and the yoga manual, are provided in other publications.^41,51^ A full course of treatment consisted of 10 GA treatments and 8 YT sessions.

#### Providers

The providers included five acupuncturists and five yoga providers. The acupuncturists were licensed in the state of NY and certified by the National Certification Commission for Acupuncture and Oriental Medicine. The yoga providers were either certified yoga therapists with the International Association of Yoga Therapists (C-IAYT) or registered yoga teachers with advanced training and extensive experience working with chronic pain populations (E-RYT-500).^52^ Acupuncturists were credentialed within MMG and IFH. As YT is not yet a licensed profession, the yoga providers were registered as volunteers, and were restricted against touching patients. Providers met all other institutional requirements.

### The implementation assessment

#### Stakeholders

The team consisted of principle investigators (PIs—B.K., M.D.M., R.T.), co-investigators (A.N., B.J.A., S.M., P.M., D.M.), and clinical research coordinators (CRCs—M.M., H.G.). The five acupuncturists were all involved with our previous clinical trial,^37^ whereas most of the five yoga providers were participating in a clinical trial for the first time. Among the investigators were acupuncture specialists (A.N., B.J.A., D.M.), yoga specialists (S.M.), and medical clinicians/researchers (R.T., B.K., M.D.M.). Stakeholders at the CHCs included the referring providers, site administrators, and clinical staff.

#### Implementation assessment tools

Several tools and methods were used to collect data to proactively address barriers and facilitators to implementation. These were developed in our previous acupuncture trial^37,44^ and consisted of regular stakeholder meetings and provider surveys.

##### Meetings

One-hour research team meetings took place weekly by videoconference. These were frequently preceded by 30 min meetings between R.T., D.M., M.M., and H.G. to discuss detailed issues associated with study operations. Yoga providers had 1-h weekly videoconference meetings with S.M., R.T., and D.M., and acupuncturists had monthly 1-h meetings with A.N. and B.J.A. These meetings were conducted from October 2018 until March 2020.

##### Provider surveys

A link to the online provider survey, housed in the Research Electronic Data Capture system at Albert Einstein College of Medicine, was embedded in the weekly intervention session schedule e-mail that was sent to all providers. The surveys were designed to facilitate implementation, and recorded information about the physical space, participant flow, participant issues, equipment, language, use of the manual/documentation, and what went well. The surveys were read by study team members and used to facilitate implementation discussions during team meetings.

## Results and Discussion

Table 1 summarizes key implementation issues within the five CFIR domains and categorizes them as barriers and facilitators to implementation.

**Table 1.** Implementation Issues as Barriers and Facilitators

CFIR domain	Barrier	Facilitator
Intervention characteristics		
Intervention sources	GA and YT not offered in CHCs	Opportunity for participants to get access to these therapies
Evidence		Well documented evidence in support of these therapies
Relative advantage		Evidence-based alternative to pharmaceutical pain treatment
Adaptability	Fidelity must be sustained to achieve expected effectiveness Requires trained and credentialed providers	Treatment manuals allow flexibility Therapies can be adapted to different environments, spaces, and schedules
Trialability		This study will facilitate future studies and real-world implementation
Complexity	Reasons for high initial complexity: Not routinely undertaken in CHCs Different theoretical constructs and professional cultures Specialist providers required	Good communication Open relationships
Design quality and packaging		Policies and procedures manual was created to capture and systematize implementation strategies
Cost	Limited insurance coverage for acupuncture Little to no insurance coverage for yoga	
Outer setting		
Patient needs and resources	Lower income/minority status and comorbidities of patient population presented additional challenges	Adaptation of therapies Bilingual staff and translation services Physician referral—increased awareness of the therapies
Cosmopolitanism	Temporary implementation initiative	Connection to health care systems
Peer pressure	These therapies are not conventionally provided in CHCs	Offering these therapies could provide a competitive advantage
External polices and incentives	Insurance reimbursement limitations for these therapies	Opioid crisis Joint Commission revised pain assessment and management standards Value-based reimbursement models
Inner setting		
Structural characteristics	Different types of stakeholders	Specialist knowledge and role of different stakeholders
Networks and communication		Consensus decision making Specific research team members engagement with CHC personnel
Culture	Different cultures associated with the three professions; biomedicine, acupuncture, and yoga	
Climate		Opioid crisis and increased receptivity to nonpharmacologic therapies Career enhancement incentive for acupuncturists and yoga providers
Characteristics of individuals		
Participants (patients)		Physician referral Information about study and therapies
Site staff		Cooperative and supportive
Research team and providers		Bilingual providers and clinical research coordinators Committed to working with underserved and improving access to these therapies
Implementation process		
Planning	Site-specific requirements	Research team meetings
Engaging		Established professional relationships with CHC staff Commitment of research team and providers
Executing	Scheduling, patient wait times, treatment fidelity, coordination of lifestyle recommendations from GA and YT, provider credentialing and orientation for working in biomedical setting, language, communication between providers	Our previous clinical trial Research team and provider meetings Provider surveys Ongoing problem solving
Reflecting and evaluating		Research team and provider meetings Provider surveys Mid project review meeting

CFIR, Consolidated Framework for Implementation Research; CHC, community health center; GA, group acupuncture; YT, yoga therapy.

### Intervention characteristics

#### Intervention source

A defining characteristic of our study was the use of therapies that are not typically used in biomedical health care settings and are therefore classified as externally developed interventions. Bundled GA and YT had never been undertaken in these settings.

#### Evidence

The effectiveness of acupuncture and yoga is supported by a significant body of research evidence. Awareness of this was facilitated by R.T. (co-PI), a primary care physician and a colleague of the referring providers.

#### Relative advantage

These therapies have a distinct advantage because they offer an evidence-based low-risk alternative to pharmaceutical pain treatment.

#### Adaptability

Different elements of the acupuncture and YT treatments were deemed core or flexible. This ensured consistent use of core elements and flexibility to adapt treatments to patient-need and different environments.

The core elements associated with GA included the group dynamic (ideally at least two participants receiving treatment at the same time), treatment according to the acupuncture manual,^44^ a defined minimum number of treatments (dosage), and delivered by providers who are appropriately trained and credentialed. Flexible aspects of GA included treatment variations defined in the acupuncture manual,^44^ the space where the treatments took place including seating configurations, the number of participants, and session length to accommodate variable numbers of participants on a given day.

The core elements of YT included breathing practices (pranayama), physical postures (asana) for mobility and stability, mental practices (dhyana and dharana), and applied philosophy. Within these categories, yoga providers were able to be flexible with which specific practices (from a select list of appropriate options) would suit the individual and how each might be modified accordingly. All participants began with diaphragmatic breathing as an essential practice and additional practices were added such that ideally at least one practice from each category would be introduced at some point during the intervention.

#### Trialability

Implementation barriers and facilitators for GA had been studied in our previous trials.^37,53–55^ This study identified additional issues associated with bundling GA with YT. These studies will facilitate future large-scale trials and real-world implementation.

#### Complexity

Introducing GA and YT to CHC settings is complex. These therapies had not been widely or consistently used in these settings before, are based on different theoretical constructs relative to each other and to biomedicine, are therapies that biomedical clinicians are not typically trained in, and require different space arrangements and scheduling structures than are typical in these settings. In addition, specialist providers needed to be hired, credentialed, and trained, and even though they are all classified as CIH providers, their training and professional cultures have important differences. Therefore, this study had a triad of different professional input—biomedical, acupuncture (East Asian medicine), and yoga.

#### Design quality and packaging

The purpose of our feasibility study was to determine the optimal design for an effectiveness/implementation hybrid study to follow. This will be facilitated by a policies and procedures manual that was created in this study.

#### Cost

There are many aspects of implementing CIH therapies, such as acupuncture and yoga, in biomedical settings that incur additional costs above what could be covered by existing infrastructure and services. These include hiring the providers, space needs, administrative support, and specialized supplies. Reimbursement to cover these costs through government or private insurance is highly variable from state to state, and across the patient population that is served by each clinic.^19^ Acupuncture is covered by many private insurers, Medicaid (in some states), and Medicare (only for chronic low back pain). Yoga has very limited insurance coverage. The lack of consistent insurance coverage for these therapies represents the most significant barrier to implementation in biomedical settings.

### Outer setting

#### Patient needs and resources

CHCs predominantly provide health care to minority and low-income patient populations. The specific needs and resources of these patients are important factors for implementation, and strategies were developed to best address these needs and their potential lack of resources. Examples that we encountered included the need for bilingual providers (English and Spanish), scheduling flexibility, sensitivity to transportation costs and barriers, challenges associated with following lifestyle recommendations (diet, exercise, stress management etc.), high incidence of multimorbidities, and the impact of low socioeconomic and/or immigrant or minority status on stress and mental health.

#### Cosmopolitanism

The CHCs are part of larger health care centers and networks. However, the impact on our project from these connections was not significant, possibly because it was an externally funded temporary implementation initiative.

#### Peer pressure

There are very few CHCs that offer CIH therapies, and so this project is quite novel and unique. If these CHCs are able to implement these therapies, following on from our research, then they would be the early adopters and would (hopefully) put peer pressure on others.

#### External policies and incentives

The primary external policies and incentives for the CHCs are mainly related to the opioid crisis and emerging payment systems for some CIH therapies. In response to the opioid crisis, the Joint Commission formulated new and revised pain assessment and management standards that require provision of nonpharmacologic therapies for pain treatment.^4,56^ Increased insurance coverage for acupuncture through the Centers for Medicare and Medicaid,^57^ and emerging value-based reimbursement models, are also important external incentives to implement acupuncture.

### Inner setting

The inner setting perspective in our externally funded study is different from an evidence-based intervention being implemented through a decision within the health care center. Therefore, the characteristics of the CHC were not a focus of our study. The inner setting in our study was about dynamics within the research team and providers, and the way we interfaced with the CHC personnel.

#### Structural characteristics

Structural complexity was evident in our study through the number of different types of people involved. These included three categories of providers (medical providers, acupuncturists, and yoga providers), researchers, and CHC personnel.

#### Networks and communication

An important aspect of our study was that decision making was largely by consensus. Regular team meetings and weekly surveys created strong feedback loops and opportunities for discussion and group decision making. Two members of the study team (R.T. and D.M.) played important roles as facilitators of cohesion between the study and the CHCs. R.T., a medical doctor within one of the CHCs, created a valuable link that benefitted the study overall. D.M. was both a provider and part of the research group, interfacing between the providers, CRCs, and CHC personnel. D.M. was instrumental in solving many logistical issues that arose, and in finding solutions to challenges associated with implementing therapies within the CHCs. The roles that R.T. and D.M. played were critical to the success of the study and implementation.

#### Culture

This study combines two overlapping but distinct health care cultures—those of biomedicine and those of CIH. As a generalization to broadly distinguish between the two, biomedicine follows a disease-focused, find it and fix it, reductionist model,^58,59^ whereas CIH therapies treat and engage patients in self-care, and follow a patient-centered holistic approach that incorporates self-efficacy and the biopsychosocial model.^60^ Although the barriers between these two approaches to health care have lessened over the past two decades, these cultural differences are not insignificant.^61^ Training of CIH providers and primary care personnel to work together in biomedical settings is an important factor in facilitating implementation.^62^

Also significant are the subtler cultural differences between acupuncture and yoga. Training to be an acupuncturist requires a minimum of 1905 h, whereas training to be a yoga therapist requires 1000 h. Much of the additional hours for acupuncturists are focused on biomedical and clinical education. Acupuncturists must pass board exams to be licensed, whereas yoga therapists are certified and there is no licensure system at this time. There are also differences in the philosophies of the disciplines, which influence approaches to patient care. These differences between the professions were evident in our study through the adaptability to working in a biomedical setting and familiarity with working with patients with significant comorbidities.

#### Climate

The opioid crisis, and the associated restrictions on prescribing opioid medications, has created increased receptivity to evidence-based nonpharmacologic approaches. This represents an opportunity for CIH providers, many of whom would like to be salaried employees in biomedical settings and/or pursue research careers. This represented an incentive for CIH providers to work in this study. The team-based nature of the study management, consensus-driven decision making, and effective feedback mechanisms created a study climate that enhanced their growth and development. The experience gained from this study would situate them more strongly for salaried positions in biomedical settings, and to further engage in research.

### Characteristics of individuals

In our study this aspect of the CFIR primarily pertained to the study participants, research team, and health care providers, and secondarily to the CHC personnel who provided patient referrals, assistance with attaining space and equipment, and scheduling facilitation. The knowledge and perspectives of the CHC personnel about the value of the therapies, beyond how that impacted referrals, was not important to successfully undertake the study, but will be if these therapies are permanently implemented. The CHC personnel were supportive of implementing these therapies and of the study in general. Medical providers appeared confident in the evidence supporting the effectiveness of the therapies and were enthusiastic about referrals.

Referral to the study likely enhanced participation, and utilization of bilingual providers and CRCs facilitated communication. All members of the study team and the providers had a commitment to working with the underserved and increasing access to these therapies.

### Process

Using the CFIR's four essential activities of implementation process (planning, engaging, executing, and reflecting and evaluating) we describe the process used to implement our feasibility study.

#### Planning

A detailed plan for our study is described in a previous publication.^41^ We used a repeated measures quasi-experimental design in which measurements were taken on participants before (when receiving usual care) and at intervals after treatment was initiated.

Each CHC site had its own unique characteristics associated with the sites' culture, usable space, competing demands upon the designated space, administrative and maintenance staff that assisted implementation, use of and access to storage space, referring providers, patient population, and acupuncture and yoga providers who were assigned to each site. This variation between the individual sites meant that planning efforts needed to be both general and site specific.

#### Engaging

Implementation depended upon engagement of both study team members (researchers and providers) and personnel at the CHCs. Study team members had a high level of engagement in successfully implementing the therapies, which was enhanced through regular meetings and other opportunities to discuss outcomes and problem solve. Facilitation of patient referrals at the CHCs was undertaken by dissemination of study information through collegial networks, discussions, electronic medical records, e-mail notifications, and flyers. This was facilitated by the relationships that R.T. and D.M. had with CHC personnel.

#### Executing

Study execution was largely undertaken by the CRCs and D.M. under the management of the research team. Regular research team and provider meetings facilitated by PIs and co-investigators were critical for executing the study plan, resolving implementation challenges, tracking study participation flow, collecting outcome data, and monitoring implementation through the provider surveys.

##### Provider surveys and meetings

Outcomes associated with the provider surveys are presented in Table 2. The implementation categories are listed in the first column. Throughout the 159 days of the trial 597 entries were recorded in the various categories, which represented feedback from 238 individual providers—104 from the acupuncturists, and 134 from the yoga providers.

**Table 2.** Provider Survey Responses

Topic	Acupuncturists			Yoga therapists		
	N	Percent of category	Percent of responses	N	Percent of category	Percent of responses
Physical treatment space/set up/break down (*n* = 49)	36	73	15	13	27	4
Workflow/patient flow/time management (*n* = 73)	41	56	17	32	44	9
Interchange or interaction with clinical staff or medical personnel (*n* = 21)	17	81	7	4	19	1
Clinical yoga or medical question I need to discuss Clinical acupuncture or medical question I need to discuss (*n* = 71)	22	31	9	49	69	14
Issues with prop use, pose modification, forms, homework sheets (*n* = 32)	N/A			32	100	9
Issues with language/communication (*n* = 6)	2	33	1	4	67	1
Issues with comorbidities (*n* = 2)	2	100	1	0		
Issues with access or patient preferences (*n* = 13)	9	69	4	4	31	1
Issues or challenges with the manual or documentation (*n* = 20)	0			20	100	6
Other clinical issues (*n* = 14)	7	50	3	7	50	2
Something that went well (*n* = 201)	70	35	29	130	65	37
Additional comments (*n* = 96)	37	38	15	59	62	17
Total No. of responses across all categories	243			354		
No. of responses by individual providers	104			134		

Percent of category was calculated across all respondents (acupuncturists and yoga therapists) for each category. Percent of responses was separately calculated for the acupuncturists and for the yoga therapists.

*N*, number of survey entries; N/A, not applicable.

Survey feedback showed some interesting trends. Participants received acupuncture first, and therefore the acupuncturists established the treatment spaces at the beginning of a session. Consequently, it is not surprising that acupuncturists submitted more entries related to the treatment space, patient flow, and interaction with CHC personnel. These issues included getting access to suitable space and setting it up, accommodating the scheduling of participants and schedule deviations (arriving early and lateness), modifying treatment time to dovetail with the yoga sessions, and feedback on needing assistance from the CHC personnel. As acupuncture requires access to body areas that may have been covered by clothing (especially in winter), the acupuncturists also reported more on this issue. Acupuncturists also reported more on patient preferences for treatment variations and minor adverse events.

The yoga providers had more entries related to questions about YT and the participant's medical condition, but the acupuncturists reported more about participant comorbidities, possibly because they have greater training in biomedicine. The yoga providers reported issues with their equipment, the manual, and documentation, whereas the acupuncturists did not report on these at all. Given that the acupuncturists had all worked in our prior trial,^37^ these issues for the acupuncture therapy had likely already been resolved. The yoga providers requested greater communication with the research team, and therefore their meetings occurred weekly, as compared to the monthly meetings for the acupuncturists.

Interestingly, the greatest number of entries were about what went well. These ranged from good patient flow, patient reports of less pain, and improved quality of life, synergy in the groups and dyads, gratitude for CHC personnel, and improvements in the manual, documentation, and equipment for the yoga providers. The greater number of survey entries from the yoga providers overall was likely related to less familiarity with being part of a clinical trial. Also, of interest in this section was greater discussion by the yoga providers about participant emotions, and the spiritual aspects of their treatments and interactions with the participants.

##### Study team meetings

Implementation issues were the most commonly discussed items in these meetings and included the following:

- Scheduling issues: It was not possible to give participants regular appointment times due to the schedule constraints defined by the study design (sequencing of acupuncture followed by yoga intake or session), managing new and ongoing participants, and participant attendance and adherence to appointment time. These were ongoing issues that improved over time through strategic scheduling and improved communication.
- Patient wait times: Minimizing wait time between acupuncture and yoga appointments was very challenging. Initially some participants had wait times in excess of 45 min, which was rectified as the trial progressed.
- Fidelity of treatments: The schedule layout mandated by the study design, combined with the necessity to minimize patient wait times, and variability of recruitment flow sometimes resulted in there only being one participant receiving acupuncture or yoga. We were concerned that this did not represent GA, or YT dyads. Dovetailing the acupuncture and yoga schedules, along with patient lateness, sometimes led to shortened acupuncture treatments, which negatively impacted treatment fidelity. As participant flow and timing improved these problems were rectified. Orientation to the yoga manual required additional oversight and coordination within the yoga team.
- Consistency of lifestyle and therapeutic instruction: Efforts were made to reduce redundancy and ensure that instruction was consistent between the acupuncturists and yoga providers to the participants.
- Preparing the acupuncture and yoga providers to work in biomedical settings and within a clinical trial: Team meetings prioritized the best ways to support provider adaptation to these different work environments. Issues of significant focus included—professionalism, communication with the CHC administrative and medical staff, security IDs, HIPAA standards, charting and documentation.
- Differences between the CHCs: Each site had unique characteristics associated with physical layout, equipment storage, access, patient waiting areas, administrative and maintenance support, and scheduling challenges. Therefore, procedures and issues often were addressed in a site-specific way.
- Multiple languages: Critical to the success of implementing these therapies within the CHCs was that members of the study team were bilingual (English and Spanish) or prepared to work through translation services. Managing the mix of participants, providers, and CRCs with regard to their language abilities was an important aspect of implementation. GA could accommodate a mix of English and Spanish speaking participants, but YT had more verbal instruction and so dyads needed to be monolingual.
- Communication between the acupuncturists and yoga providers: Communication was important given the need for tight coordination between the acupuncture and yoga sessions.

#### Reflecting and evaluating

The study team and provider meetings provided a lot of opportunity for evaluation and reflection as the study proceeded. These meetings led to constant implementation process tweaking, which occurred more frequently in the trial's early stages. At the mid-point of the study an especially important research team meeting took place with the aim of determining whether the current approach needed any significant changes to ensure that the study accurately represented what could be feasibly implemented in a real-world setting, including fidelity of the therapies. The issues that were discussed at this meeting included: scheduling issues, lack of sufficient participants to form a group for GA and yoga dyads, and reduced acupuncture treatment times.

## Conclusions

Our feasibility trial and the use of the CFIR structure provided a meaningful and pragmatic way to identify barriers and facilitators associated with implementing bundled GA and YT in CHC settings. The fact that it was a clinical trial did not significantly detract from the ability to focus on implementation issues. This approach may represent a valuable way to undertake a small-scale implementation study, and also explore real-world effectiveness. Outcomes of such studies can produce evidence to be leveraged for larger scale implementation studies.

Important implementation issues that we identified were related to both practical considerations and theoretical/philosophical distinctions between the two therapies. Practical considerations like space, storage, and scheduling required flexibility and adaptability from both the CHCs and CIH providers. Bringing together two CIH therapies introduced its own set of challenges. The therapies had to be modified to be used together such that the core aspects of each were sustained to maintain treatment fidelity. Although some implementation issues were associated with differences between the three CHC sites, these were mainly related to the physical setting and management of the CHC, such as room configuration and scheduling methodology. Therefore, our study did not identify implementation issues that could be ascribed to specific types of CHC settings, and we anticipate that our findings are likely to be relevant to most CHCs.

Based on this trial we are planning a larger scale effectiveness/implementation hybrid study. The policies and procedures manual that was created from this feasibility study will be used to guide the hybrid study, which will require a greater emphasis on implementation-specific outcomes. These could include education-initiated physician referral rates, participant treatment completion rates, and qualitative studies undertaken with referring providers, site administrators, providers, and participants. Cost effectiveness is another important implementation issue, and evaluations of this would also be a valuable area of investigation.
